# An investigation of the perceptions of laboratory animal welfare issues among undergraduate and graduate veterinary students in southeastern China

**DOI:** 10.3389/fvets.2023.1335484

**Published:** 2024-02-12

**Authors:** Shihong Yan, Hongyang Li, Jin Lin, Huimin Chen, Shasha Liu, Hongxiu Diao

**Affiliations:** ^1^Key Laboratory of Animal Pathogen Infection and Immunology of Fujian Province, College of Animal Sciences, Fujian Agriculture and Forestry University, Fuzhou, China; ^2^Joint Laboratory of Animal Pathogen Prevention and Control of Fujian-Nepal, College of Animal Sciences, Fujian Agriculture and Forestry University, Fuzhou, China

**Keywords:** Chinese veterinary students, animal welfare, laboratory animal, university education, survey research

## Abstract

Animal experiments have played a significant role in advancing scientific knowledge and enhancing people's quality of life. In order to better understand the opinions and knowledge of veterinary students in the domain of laboratory animal welfare and to explore and advance the teaching methods used in animal ethics education, a questionnaire was designed and used to conduct a survey among undergraduate and postgraduate students majoring in veterinary medicine. The survey encompassed various topics, such as students' level of knowledge about animal welfare, their perspectives on laboratory animals, their proficiency with animal experiments, and their opinions on teaching methods and content. The respondents were a total of 150 undergraduate students and 148 graduate students. The survey results indicated that most students expressed a strong sense of responsibility for the safeguarding of the welfare of experimental animals. However, there were a few students who lacked compassion for animals. Additionally, there was a general lack of basic theoretical knowledge of animal ethics and an inadequate grasp of experimental techniques among current students. Furthermore, most of the participants expressed a strong sense of responsibility to advocate for animal welfare. Although a substantial number of students were unaware of the existence of agencies for the supervision of work involving laboratory animals, they supported teaching and supervision in the domain of animal welfare and were open to various teaching methods and topics of content. In conclusion, targeted training and education regarding laboratory animal welfare and ethics should be conducted in the future to address the specific needs of students. This study provides a foundation for future animal welfare education and will help to improve the professional skills and humanistic qualities of veterinary students.

## 1 Introduction

In recent decades, there has been remarkable worldwide advancement in the understanding of diseases and their diagnosis and treatment. This progress is largely attributable to the introduction and utilization of laboratory animals. Animal experimentation represents one of the primary methods used to discover new knowledge in biomedical science, with experimental animals consistently playing a vital role in the new stages of the rapid development of research in precision medicine in China. Through animal experiments, researchers can delve deeply and comprehensively to explore the mechanisms underlying the pathogenesis and progression of human or animal diseases (1, 2). Additionally, animals have been utilized to enhance our understanding of animal and human anatomy and physiology, as well as to assess the safety and efficacy of drugs and vaccines (3–5). In short, experimental animals are indispensable in multiple areas of life science and have made significant contributions to the advancement of scientific knowledge and enhancement of people's quality of life. Although most people understand and appreciate the need for animal-based research, the welfare of laboratory animals and the ethics of their use has become a recognized issue and widespread concern worldwide (6–8).

The progress of society and national development in quality of life have led to a paradigm shift in the perception of animals. Animals are no longer considered mere extensions or tools of human beings, but rather are regarded as independent beings with the inherent right to life (9, 10). This recognition of the importance of animal welfare and a growing awareness of the principles of animal rights have resulted in a heightened emphasis on the ethical considerations associated with the use of experimental animals (11). Consequently, increasing amounts of attention are being paid to the ethical issues surrounding experimental animals.

Ethical issues surrounding experimental animal research extend beyond the wellbeing of the animals themselves; they also impact the scientific integrity of experimental outcomes (12). Furthermore, these ethical considerations have implications for the quality of education and experience with scientific research for veterinary students. It has been demonstrated through various studies that the attitudes of researchers toward laboratory animals can be influenced by a range of psychosocial factors, including their level of education and training (13, 14). In order to obtain reliable and effective experimental results, it is crucial to prioritize the welfare of laboratory animals, which is directly dependent on the actions of the experimenter (15). As a result, university education plays a pivotal role in enhancing the theoretical knowledge and practical skills of veterinary students in the domain of laboratory animal welfare and ethics, enabling them to ensure that the welfare of laboratory animals is maintained and their needs guaranteed to be met in a timely and effective manner.

Despite the emphasis placed on animal welfare by leading veterinary organizations in China with well-defined standards, unfortunately there exists a clear disparity between the southeast regions and “first-tier” cities like Beijing in terms of veterinary student education and training in animal welfare (16).

The purpose of this study was to assess current perceptions and understanding of laboratory animal welfare among undergraduate and graduate students of veterinary medicine in southeastern China. The investigation mainly focused on evaluating students' levels of knowledge regarding animal welfare, their proficiency with experimental techniques, and their opinions on approaches to teaching animal welfare. Additionally, we analyzed existing educational practices and identified challenges related to the welfare of and ethical issues surrounding laboratory animals in colleges and universities. Based on our survey results, we aim to optimize education and training on experimental animal ethics for veterinary students. This will enhance the quality of teaching about animal welfare and subsequently promote the professional competence of future veterinarians.

## 2 Materials and methods

A self-designed questionnaire was used to evaluate the current opinions of university students on laboratory animal welfare. Different kinds of questions were included: responses were given in multiple-choice form (including questions with single-answer choices and with single- or multiple-answer choices) or on a Likert scale. The questionnaire consisted of similar content to that of other laboratory animal studies in China and abroad (17–19), ensuring comprehensive coverage. The survey had no time restrictions, but respondents were expected to complete it within 10 min. The questionnaire was handed to the students by the head teacher of the class after a class meeting. Anonymous participation was ensured, and only undergraduate (senior and junior) and graduate students of veterinary medicine, who have direct contact with experimental animals, were targeted. No specific incentives were offered.

The questionnaire was divided into four sections: (1) the respondent's understanding of concepts related to animal welfare and ethics; (2) the current status of education on laboratory animal ethics; (3) attitude toward laboratory animals and mastery of animal experimentation skills; and (4) opinions on teaching and awareness of topics surrounding the welfare of and ethics issues relating to laboratory animals.

All data were imported into a Microsoft Excel spreadsheet (Microsoft Corporation, Redmond, WA). A total of 300 surveys were collected, of which 298 surveys were included in the analysis, comprising responses from 150 undergraduates and 148 graduate students. The response rate was 99.3%. Descriptive statistics for all variables of interest were tabulated in Excel. One of the authors individually screened each survey to calculate the percentage (%) of questions answered.

This study received approval from the Ethics Committee of Scientific Research at Fujian Agricultural and Forestry University. Respondent information has been anonymized to ensure confidentiality.

## 3 Results

### 3.1 Awareness of animal welfare and ethics

Table 1 presents a summary of the responses regarding student awareness of animal welfare. Among undergraduate students, 86.7% were aware of the concept of animal welfare and ethics, while among graduate students, this proportion was 72.9%. However, only 26.0% of undergraduates and 11.5% of graduates were familiar with the “Five Freedoms” and “3Rs principle” in animal welfare. It was observed that many undergraduate students had heard of these concepts but were unfamiliar with their specific content. Moreover, 32.6% of undergraduates and 48.7% of graduates reported having no prior knowledge of animal welfare, indicating a general lack of awareness among students regarding animal welfare and ethics.

**Table 1:** Level of understanding of concepts relating to animal welfare and ethics.

**Question**	**Response**	**No. of respondents (% of survey sample)**	
		**Undergraduate students**	**Graduate students**
Are you familiar with the concept of animal welfare and ethics?	Yes	130 (86.7%)	108 (72.9%)
	No	20 (13.3%)	40 (27.1%)
Do you know of the concepts of the “3Rs principle” and the “Five Freedoms” in animal welfare?	Yes, know well	39 (26.0%)	17 (11.5%)
	Yes, but don't know the details	62 (41.3%)	58 (39.2%)
	No	49 (32.6%)	73 (48.7%)

### 3.2 Status of ethics education received on the utilization of experimental animals

Among the respondents, a majority of undergraduates (85.3%) and graduate students (87.9%) thought that they lacked knowledge of animal ethics in relation to laboratory animals. However, a considerable proportion of undergraduates (68.7%) and graduate students (60.1%) still expressed the intention of giving due consideration to the welfare of laboratory animals during animal experiments. Furthermore, most of the respondents believed that the teaching of laboratory animal ethics would be beneficial to their future careers. Only a small proportion of undergraduates (2%) and postgraduate students (4.7%) believed that such teaching would not be helpful to their future careers (Table 2). These findings suggest that majority of students demonstrated recognition of the relevant ethical issues and a willingness to participate in education on laboratory animal ethics.

**Table 2:** Opinions on the status quo of education received on laboratory animal ethics.

**Question**	**Response**	**No. of respondents (% of survey sample)**	
		**Undergraduate students**	**Graduate students**
Do you think you lack knowledge of animal ethics in relation to laboratory animals?	Yes	128 (85.3%)	131 (87.9%)
	No	22 (14.7%)	17 (12.1%)
Will you pay more attention to the welfare of experimental animals in the process of conducting experiments?	Yes	103 (68.7%)	89 (60.1%)
	No	47 (31.3%)	59 (39.9%)
Do you think education on laboratory animal ethics will be helpful for your future career?	Yes	147 (98.0%)	141 (95.3%)
	No	3 (2.0%)	7 (4.7%)

### 3.3 Attitude toward experimental animals and mastery of skills in animal experimentation

Table 3 presents an overview of the responses to multiple survey questions relating to animal experiments. When dissecting animals or performing other experiments on injured animals, 14.7% of undergraduate students and 12.2% of graduate veterinary medicine students expressed adopting a similar mindset to that adopted when performing physical or chemical experiments. A majority of undergraduate students (61.3%) and graduates (75.0%) reported that although they did not show their emotions during animal experiments, they were emotionally affected. Furthermore, 24.0% of undergraduates and 12.8% of graduates expressed difficulty in performing experiments on injured animals. Regarding upholding of the “humanitarian spirit” during experimental procedures on animals, 19.3% of undergraduate students believed that they did not uphold this principle at all. Most of the students (66% of undergraduates and 81.8% of graduates) indicated that while they aimed to maintain the humanitarian spirit, their skills in experimental animal procedures did not meet the relevant standards. Only a small percentage of undergraduate students (6.0%) and postgraduates (10.1%) reported both upholding the humanitarian spirit and complying with technical specifications. The results of these two components of the survey demonstrate that most of the students prioritized the welfare of laboratory animals and strove to adhere to the guidelines for animal experiments. However, there appeared to be a general lack of proficiency in experimental techniques among current students, with only a small number lacking a humane approach.

**Table 3:** Attitudes toward experimental animals and mastery of experimental skills.

**Question**	**Response**	**No. of respondents (% of survey sample)**	
		**Undergraduate students**	**Graduate students**
How do you feel when you dissect animals or perform other experiments that cause injury to animals?	I adopt the same mindset as for physical or chemical experiments	22 (14.7%)	18 (12.2%)
	Although I don't show it, I still feel something	92 (61.3%)	111 (75.0%)
	It feels difficult to perform these experiments	36 (24.0%)	19 (12.8%)
Is humanitarianism maintained during animal experiments?	I feel nothing at all	29 (19.3%)	12 (8.1%)
	Yes, but the procedures are not standard	112 (74.7%)	121 (81.8%)
	Yes, the procedures are standard	9 (6.0%)	15 (10.1%)

### 3.4 Opinions on teaching and advocacy relating to laboratory animal welfare and ethics

Table 4 presents the findings from the section of the survey on teaching and advocacy relating to laboratory animal welfare and ethics. The results indicated that a significant majority of undergraduate (96.7%) and graduate (98.6%) students in China acknowledged their responsibility to advocate for and implement measures ensuring animal welfare. Moreover, a high proportion of students (specifically, 94.7% of undergraduates and 98.6% of graduates) believed that incorporating animal welfare knowledge and methods into the current teaching curriculum would be crucial. These results showed that most of the students accepted the provision of education on animal experiments and supported the ethical supervision of animal experiments. However, most of the students, particularly undergraduates (81.3%), were unaware of the existence of the Ethics and Animal Welfare Committee. Despite this, the majority of undergraduate (92.0%) and graduate (94.6%) students still expressed their support for the supervision of experimental animal welfare in teaching and research, highlighting the students' willingness to prioritize animal welfare and the need for further education and awareness regarding ethical considerations relating to laboratory animal experiments.

**Table 4:** Opinions on teaching and advocacy relating to laboratory animal welfare.

**Question**	**Response**	**No. of respondents (% of survey sample)**	
		**Undergraduate students**	**Graduate students**
Do you consider yourself responsible for advocating for and implementing measures promoting animal welfare in our country?	Yes	145 (96.7%)	146 (98.6%)
	No	5 (3.3%)	2 (1.4%)
Under the current teaching curriculum, do you think it is necessary to teach knowledge and skills relating to animal welfare?	Yes	142 (94.7%)	146 (98.6%)
	No	8 (5.3%)	2 (1.4%)
Do you think that teaching or scientific research related to laboratory animals requires supervision by an animal welfare regulator?	Yes	138 (92.0%)	140 (94.6%)
	No	12 (8.0%)	8 (5.4%)
Are you aware of the existence of the Ethics and Animal Welfare Committee?	Yes	28 (18.7%)	45 (30.4%)
	No	122 (81.3%)	103 (69.6%)

Table 5 presents the survey results indicating students' preferences and priorities regarding different teaching methods and content relating to laboratory animal welfare and ethics. Among undergraduate students, the highest proportion (83.3%) were willing to accept alternative teaching methods for experimental skills. This was followed by the degree of acceptance of teaching based on computer simulations (76.7%), specimens (67.3%), and videos (46%). Only a small percentage of students (2%) expressed unwillingness to accept any of these alternative methods. Among graduate students, the most widely preferred teaching method was model-based teaching, with 58.1% of respondents expressing this preference. This was followed by teaching based on specimens (45.3%), computer simulations (41.2%), and videos (36.5%). It is worth noting that a larger proportion of graduate students (26.4%) compared to undergraduates expressed unwillingness to accept alternative methods. Regarding curriculum content, both undergraduate and graduate students considered the practical application of laboratory animal welfare to be the most important area of knowledge within laboratory animal ethics, with this opinion expressed by 91.3% and 94.6% of respondents, respectively. This was followed by discussion of ethical issues relating to laboratory animals (regarded as important by 81.3% of undergraduates and 72.0% of graduates), study of the relevant laws and regulations (71.3% of undergraduates, 77.7% of graduates), and the differential treatment of different species (68.6% of undergraduates, 62.2% of graduates). Only a very small percentage of students (0.7% of undergraduates, 2% of graduates) stated that they did not consider any of this content to be important. Overall, the survey findings indicated the students' willingness to explore alternative teaching methods and their recognition of the importance of practical applications, ethics discussions, laws and regulations, and species-specific considerations in education on laboratory animal welfare and ethics.

**Table 5:** Opinions on teaching and advocacy relating to laboratory animal welfare (multiple-choice questions).

**Question**	**Response**	**N. of respondents (% of survey sample)**	
		**Undergraduate students**	**Graduate students**
Which of the following laboratory teaching alternatives would you accept or advocate for?	Teaching by model	125 (83.3%)	86 (58.1%)
		Specimen-based teaching	101 (67.3%)	67 (45.3%)
		Computer simulation teaching	115 (76.7%)	61 (41.2%)
		Video-based teaching	69 (46.0%)	54 (36.5%)
		Would not advocate for any of these alternatives	3 (2.0%)	39 (26.4%)
If you want to learn about laboratory animal ethics, which of the following do you think is the most important?	Discussion of ethical issues surrounding laboratory animals	122 (81.3%)	108 (72.0)%
		Practical application of the principles of laboratory animal welfare	137 (91.3%)	140 (94.6%)
		Differential treatment of different species	103 (68.6%)	92 (62.2%)
		Relevant laws and regulations	107 (71.3%)	115 (77.7%)
		No important content	1 (0.7%)	3 (2.0%)

## 4 Discussion

Animal experiments play a crucial role in learning and skill development among undergraduate and graduate students studying veterinary medicine. However, these experiments also raise concerns regarding the welfare of and ethics of the use of laboratory animals (20, 21). It is therefore essential for students to gain a comprehensive understanding of, and to actively incorporate, measures to ensure the welfare of these animals during their university education. Doing so not only fosters their sense of empathy and compassion but also cultivates their professional competence in this domain.

Veterinary students are expected to make a strong commitment to animal welfare and demonstrate a sense of responsibility in relation to the treatment of animals. Research has indicated that veterinarians play a crucial role in identifying incidents of animal cruelty and domestic violence (13, 22–24). In the section of the survey on recognition of animal welfare and ethics, it was observed that while most respondents claimed to be familiar with these concepts, their knowledge of internationally recognized animal welfare standards was limited.

The 3Rs principle (Replacement, Reduction, and Refinement) provides ethical guidelines for the assessment and regulation of animal experimentation (25). This principle, which has been incorporated into guidelines and laws, ensures that animal experimentation meets both ethical and scientific criteria (26). The Five Freedoms (freedom from hunger and thirst; freedom from pain, injury, and disease; freedom from discomfort; freedom from fear and distress; and freedom to express normal behavior) established the five domains of animal welfare in the early 1990s and are now well recognized as highly influential in the animal welfare arena (27–29). The findings of this study indicate that a significant number of respondents were unfamiliar with the 3Rs principle and the Five Freedoms. This suggests that students generally lack a comprehensive understanding of animal welfare and ethics, highlighting the need for veterinary education programs to provide detailed instruction on these fundamental concepts, particularly for graduate students. In contrast, the results of a previous study with Italian students showed that respondents considered their own level of knowledge on the topic of animal welfare to be good (30).

Understanding the attitudes and perceptions of veterinary students in relation to animal welfare is fundamental in assessing the effectiveness and adequacy of their education (31, 32). The results of the current study indicate that students generally recognize the importance of education in laboratory animal ethics for their future careers and express a willingness to prioritize welfare issues during the development of experimental skills. In this study and in results presented by Pirrone et al. (13), it can be seen that the majority of students are open to receiving education in laboratory animal ethics, both to enhance their professional skills and to enable them to uphold humanitarian values.

It is important to note that while the majority of undergraduate and graduate students demonstrate compassion and care for experimental animals, there are still a small number of students who view them merely as tools for learning, adopting an apathetic attitude and lacking awareness of animal welfare and ethics. This highlights the need for further education and training to instill a stronger sense of empathy and ethical responsibility in these students.

Furthermore, the use of non-standard techniques and practices in laboratory animal experiments by some students is concerning. It is crucial for both undergraduate and graduate students to receive adequate training in experimental procedures in animals in order to minimize harm to animals and ensure the successful completion of experimental research. These issues are notable because these veterinary students represent future industry stakeholders who will play a role in addressing laboratory animal welfare issues and finding solutions to various welfare challenges in animal research (33, 34). One of the most common barriers to animal welfare mentioned in a previous survey is a perceived lack of researcher support to employ appropriate techniques (35). Therefore, both undergraduate and graduate students should receive better training in experimental procedures in animals; not only would this reduce animal suffering, but it would also provide them with a crucial foundation for performing successful experimental research (36, 37).

Veterinarians have a professional and ethical obligation to prioritize and promote animal welfare (38). As future practitioners in the veterinary industry, most veterinary students recognize and embrace this responsibility and express a strong sense of responsibility to advocate for animal welfare. They understand the importance of preventing harm to animals and protecting their welfare (39). To address these issues, various bodies such as Ethics and Animal Welfare Committees (AECs) and Animal Care and Use Committees (ACUCs) have been established in developed countries (40, 41). These committees evaluate research projects conducted by authorized institutions and provide reasoned opinions on them, weighing the potential human benefit against the harm caused to animals (40, 42). However, in China, the development of such bodies is still in progress, and there is room for improvement. This survey showed that, although most of the students were unaware of the existence of the Ethics and Animal Welfare Committee, they still recognized the importance of supervision. This indicates the pressing need to strengthen the publicization and awareness of these ethics committees in Chinese colleges and universities. Alternatively, it may be beneficial to establish subsidiary committees, such as a welfare supervision committee at the department or student level, to strengthen the supervision of animal welfare in daily teaching and scientific research activities. This would allow for better monitoring and implementation of animal welfare standards in research and education practices in China.

At present, there are various alternative methods of teaching that can be used in place of traditional animal experimentation, such as teaching based on models, computer simulations, specimens, or videos, among other methods (43, 44). Among these, model-based teaching, as the method that undergraduates and postgraduates are most receptive of, warrants support and promotion within university education. However, it is worth noting that a significant proportion of graduate students in this study expressed unwillingness to accept alternative teaching methods, which aligns with the findings of previous studies (45). Upon further investigation, it was found that these students believed that traditional teaching methods helped them to remember new knowledge and technological skills more easily. As a result, these graduate students suggested that a combination of traditional training methods and alternative approaches should be used to achieve the best learning results (46). In terms of the content of teaching materials, the practical application of laboratory animal welfare principles was considered by the students to be the most important component of knowledge of laboratory animal ethics. Additionally, most students deemed the discussion of ethical issues relating to laboratory animals and relevant laws and regulations to be important, and these topics should be included in future teaching curricula (47, 48).

The responses to the questionnaire highlighted the importance of education on laboratory animal ethics and revealed the overall views of veterinary students on laboratory animal welfare and ethics. The survey also identified a general lack of awareness among students regarding knowledge of laboratory animal ethics and relevant technology, laws, and regulations. This gap has been highlighted in other surveys as well, indicating the need for further training and awareness campaigns covering the scientific, legal, and ethical facets of laboratory animal research (30). It is apparent that the responsibilities of future veterinarians extend beyond the diagnosis, treatment, and prevention of animal diseases. They also have a crucial role to play as experts in and advocates for animal welfare and ethics. Therefore, both teachers and students should prioritize the implementation of the 3Rs principle in animal experimentation and acquire advanced technical skills and knowledge relating to laboratory animals. This is important not only for the personal growth and development of students but also for their ability to conduct high-quality scientific research and to progress in their future careers.

## 5 Conclusion

In conclusion, it is critical for veterinary students to be exposed to education in laboratory animal welfare throughout their undergraduate and graduate studies. Regardless of the specific course, any education related to animals should include the development of awareness of the importance of protecting the rights and welfare of laboratory animals. Universities should prioritize strengthening formal education on topics such as animal medicine, animal surgery, and the technical skills necessary to perform procedures. The aim of these efforts is to cultivate appropriate professional attitudes and ethical practices and the necessary knowledge and skills to ensure safe and effective practices among veterinary students. By integrating laboratory animal welfare education into the overall learning process, we can better prepare future veterinarians to prioritize the wellbeing and ethical treatment of all animals in their care.

## Data availability statement

The original contributions presented in the study are included in the article/supplementary material, further inquiries can be directed to the corresponding author.

## Ethics statement

This study was approved by Ethics Committee of Scientific Research of Fujian Agricultural and Forestry University, (Permit Number 111421143). All surveys were carried out according to the regulations and the respondent information is anonymized. The studies were conducted in accordance with the local legislation and institutional requirements. Written informed consent for participation was not required from the participants or the participants' legal guardians/next of kin in accordance with the national legislation and institutional requirements.

## Author contributions

SY: Writing — original draft. HL: Writing — original draft, Investigation. JL: Investigation, Writing — original draft. HC: Data curation, Writing — original draft. SL: Writing — review & editing. HD: Writing — review & editing.
